# Perusal of Newly Developed Diffuse Sclerosing Papillary Thyroid Carcinoma During Follow-up of Benign Nodules in Thyroidology

**DOI:** 10.1055/a-2858-9794

**Published:** 2026-05-15

**Authors:** Ilker Sengul, Demet Sengul

**Affiliations:** 1 Thyroidology Unit 485533 Giresun University Faculty of MedicineGiresunTurkey; 2 Endocrine Surgery 485533 Giresun University Faculty of MedicineGiresunTurkey; 3 General Surgery 485533 Giresun University Faculty of MedicineGiresunTurkey; 4 Pathology 485533 Giresun University Faculty of MedicineGiresunTurkey

Dear Editor,

We read with profound interest the article by Goryo et al. on the longitudinal ultrasonographic evolution of diffuse sclerosing papillary thyroid carcinoma (DS-PTC), published in volume 11 of *Ultrasound International Open* (R. Goryo et al., *Ultrasound Int Open* 2025;11:a27414863), in thyroidology (V. Cantisani et al., *Ultraschall Med* 2025;46:14–35). This clinicopathological variant represents a formidable diagnostic challenge, historically characterized by its aggressive behavior and extensive lymphatic involvement. The authors’ documentation of the transition from a benign-appearing thyroid architecture to overt malignancy provides a rare and invaluable glimpse into the preclinical evolution of this subtype. As such, the study underscores a critical diagnostic lacuna: although DS-PTC accounts for a minority of PTC cases, its preoperative sonographic accuracy is strikingly low at 32.4%. By identifying multiple punctate echogenic foci (MPEF) as a potential early harbinger of DS-PTC, the authors provide clinicians with a tangible diagnostic anchor. This is particularly salient given that DS-PTC often emerges within a background of chronic lymphocytic thyroiditis, which may act as a precursor rather than a mere coexisting condition. Perhaps, the most compelling observation is the rapid intrathyroidal dissemination of the tumor despite a low Ki-67 labeling index (<5%). This paradox suggests that the clinical aggressiveness of DS-PTC is not driven by intrinsic cellular proliferation, but rather by an exceptional propensity for lymphatic permeation. The findings in Case 2, in which bilateral involvement occurred within a remarkably short interval, reinforce the hypothesis that the lymphatic system serves as a high-speed conduit for tumor cell dissemination. Furthermore, this report necessitates a paradigm shift in how we monitor patients with “benign” nodules in the context of thyroiditis. Moreover, the appearance of MPEF should no longer be viewed in isolation but as a red flag for a rapidly evolving malignant process. Goryo and colleagues (R. Goryo et al., *Ultrasound Int Open* 2025;11:a27414863) have successfully bridged a gap in our understanding of the chronological progression of DS-PTC, offering a window for earlier surgical intervention in a patient population that is typically younger and prone to advanced disease at presentation. In recapitulation, the identification of MPEF as a precursor to macroscopic DS-PTC serves as a vital diagnostic milestone. Future multicenter studies should now focus on validating these sonographic markers to refine our early detection protocols and potentially mitigate the morbidity associated with the advanced stages of this aggressive variant. This issue merits further investigation. We thank Goryo et al. (R. Goryo et al., *Ultrasound Int Open* 2025;11:a27414863) for their worthy study on diffuse sclerosing PTC for thyroidologists in *Ultrasound Int Open* .



## **Data Availability Statement**

The datasets used and/or analyzed during this study are available from the corresponding author upon reasonable request.

